# Storage Stability of *Arauco* Virgin Olive Oil: Evolution of Its Quality Parameters and Phenolic and Triterpenic Compounds under Different Conservation Conditions

**DOI:** 10.3390/plants12091826

**Published:** 2023-04-29

**Authors:** Romina P. Monasterio, Eduardo Trentacoste, Carlos López Appiolaza, María Gemma Beiro-Valenzuela, Irene Serrano-García, Lucía Olmo-García, Alegría Carrasco-Pancorbo

**Affiliations:** 1 Instituto de Biología Agrícola de Mendoza (IBAM), UNCuyo-CONICET, Facultad de Ciencias Agrarias, Alt. Brown 500, Mendoza 5505, Argentina; clopezappiolaza@mendoza-conicet.gob.ar; 2 Department of Analytical Chemistry, Faculty of Sciences, University of Granada, Ave. Fuentenueva s/n, 18071 Granada, Spain; gemabv@ugr.es (M.G.B.-V.); iserrano@ugr.es (I.S.-G.); luciaolmo@ugr.es (L.O.-G.); alegriac@ugr.es (A.C.-P.); 3 Instituto Nacional de Tecnología Agropecuaria (INTA), Estación Experimental Agropecuaria La Consulta, Ex Ruta 40 km 96, Mendoza 5567, Argentina; trentacoste.eduardo@inta.gob.ar

**Keywords:** storage conditions, olive oil quality parameters, phenolic compounds, triterpenic acids, storage conditions, olive oil aging markers

## Abstract

The storage conditions are very critical to minimize hydrolytic and oxidative reactions of virgin olive oils (VOOs). These reactions are logically influenced by the composition of the VOO, so that each variety may have a specific behavior. The aim of this study was to evaluate changes in quality parameters and in the phenolic and triterpenic profile of *Arauco* VOOs, a unique local variety from Argentina, after storage under different conditions. The effects of exposure to light (darkness and light), temperature (24 and 40 °C), packaging material (polyethylene (PET) and dark glass), and headspace (air and N_2_ atmosphere) were investigated for 76 days. A reduction in total phenolic compounds was observed after storage treatments, but all samples still complied with the EFSA health claim after the different handlings. Overall, the results revealed that the preservation of the oils in PET appears adequate, with improved stability when N_2_ was used in the headspace, along with darkness and low temperature. The study of phenolic profiles showed that substances previously reported as possible markers of olive oil aging, such as hydroxytyrosol and an isomer of decarboxymethyl oleuropein aglycone, also have a similar behavior during the aging of *Arauco* variety oil. Interestingly, some evidence was found that another oleuropein-derived compound (oleuropein aglycone isomer 3) could also be used as an aging marker.

## 1. Introduction

The typical aroma, taste, color, and flavor of virgin olive oil (VOO) distinguish it from other edible vegetable oils. Its characteristic flavor is a product of its complex composition, which is influenced by different factors, such as pedoclimatic conditions, botanical variety, and the technological process used during its extraction and shelf life [1,2]. Hydrolytic and oxidative reactions are the two principal and unavoidable reactions that produce a negative effect on VOO quality [3]. According to previous reports, the quality changes during storage are conditioned by its initial composition of antioxidants and fatty acids and by exposure to oxidative factors, such as light and oxygen [3,4,5].

It is of great importance to the VOO industry to preserve the olive oil’s positive attributes during the time elapsing from production to bottling and then to purchase and consumption. Therefore, choosing appropriate types of containers and storage conditions is of vital importance [6,7]. Indeed, inadequate storage conditions may cause the qualitative characteristics of the product to vary to such an extent that they may differ from those indicated on the label, which, as established by law, must contain the analytical characteristics of the oil at the time of bottling. Thus, any research that evaluates the magnitude of alterations that an oil undergoes during storage, comparing the changes that occur under the different possible conditions, can provide very useful information. The shelf life of VOO is conditioned by its initial composition; therefore, oils coming from different olive cultivars may have distinct behaviors, depending on their typical botanical and compositional characteristics [8,9].

With regard to the composition of VOO, it is known that it can be divided into two fractions: a saponifiable fraction (near to 98% of total content) and an unsaponifiable fraction (the remaining 2%). The first consists principally of triacylglycerides, and the second one (the so-called minor fraction) contains several chemical families such as phenolic compounds and pentacyclic triterpenes, among others [10,11]. Among phenolic compounds, secoiridoid derivatives are noteworthy for their high concentration. These compounds are formed by enzymatic conversion of oleuropein and ligstroside mainly during crushing and malaxation of the olive paste [11,12]. This process involves the action of endogenous enzymes, such as methylesterases and *β*-glucosidases, which hydrolyze ligstroside and oleuropein to generate the corresponding aglycone isomers [11,13]. The significance of phenolic compounds of VOO is indisputable since they are responsible for some of its healthy properties and contribute to a large extent to the organoleptic quality of the oil and to its stability. This is why they have been considered in many studies focused on storage conditions.

Table 1 presents a summary with different investigations on the stability of olive oil under various conditions. As can be seen, studies have been carried out on different single-varietal VOOs, and it has been observed that, for example, after one year of conditions such as those found in a supermarket line, the increase in simple phenolic compounds, such as hydroxytyrosol (HTY), is significant and could be therefore used as an indicator of freshness or aging [11,14,15,16,17]. As expected, during transport and storage, the samples are exposed to radiation and temperature, conditions that deteriorate the oil. However, as already mentioned, not all varieties behave in the same way. For example, a study on the varieties *Istrica belica* and *Leccino* showed that the *Istrica* variety was more stable to heating processes than *Leccino* according to different parameters [18]. Some studies have shown that *Arbequina* was less stable than *Picual* and *Hojiblanca* during exposure to high temperatures of transport and storage (35 °C) but more stable to photooxidation, again demonstrating inter-cultivar variability [9].

However, this behavior has not been studied for the *Arauco* variety, the only local variety from Argentina that has been recognized in the World Catalog of Olive Varieties [19]. *Arauco* is mainly present in old plantations with traditional management (i.e., low density and manual harvest) [20]. In Argentina, only a small number of varieties are used commercially. In traditional olive groves, generally planted before 1990, the presence of the local variety, called *Arauco*, also known as “Criolla”, is very prominent. This variety occupies 50% of the area of traditional olive groves, while 20% is occupied by *Farga*, *Empeltre*, *Manzanilla*, *Frantoio*, and *Arbequina* among others; the rest is represented by unknown varieties [21]. According to IOC statistical of international market, Argentina is the largest producer of the IOC members of the American continent, proving about 50% of the olive oil, which represents 1% of the world’s production [22]. *Arauco* was normally used for the production of table olives, but now, thanks to recent studies, the high quality of its oil in terms of fatty acid profile and antioxidant compounds has become evident [23,24]. Indeed, previous reports have found that *Arauco* VOO has the highest concentration of phenolic compounds compared with other cultivars from the same geographical area [24,25].

**Table 1:** Summary of the experimental conditions, the samples studied, and the parameters and compounds determined in very interesting previously published studies on olive oil storage.

Type and Number of Samples	Olive Varietal	Conditions Studied	Components Studied and Parameters Evaluated	Ref.
160 commercial EVOOs from Spain	n.i.	**Time:** 12 months **Conditions:** darkness; 20 °C **Type of container:** n.i.	Phenolic compounds LC-MS	[11]
One commercial EVOO from Tunisia	n.i.	**Time:** 0 to 12 months **Condition:** diffused light (type: n.i.); RT **Type of container:** stainless, jar, clear PET, clear and dark glass	Quality parameters; fatty acids analysis (GC-FID); pigments (spectrophotometrically); total phenols (Folin-Ciocalteau)	[26]
14 commercial EVOOs from Italy	n.i.	**Time:** 11 weeks **Conditions:** 12 h of light (600 lx) per day (type: n.i.); 22 °C **Type of container:** green glass 750 mL	Quality parameters; volatile compounds (HS-SPME-GC/MS); chlorophyll content (spectrophotometrically); α-tocopherol and phenolic compound (LC-DAD-FLD); phenolic oxidation products (LC-MS)	[4]
Two commercial EVOOs from Italy	n.i.	**Time:** 10 months **Conditions:** 12 h of light (500 lx) per day (type: LED; 380 to 780 nm); 25 °C **Type of container:** green glass, ultraviolet grade absorbing glass, multilayer (plastic-coated paperboard aluminium foil)	Quality parameters; α-tocopherol (LC-DAD-FLD); phenolic compounds (LC-DAD); volatile compounds (HS-SPME-GC-MS); sensory analysis	[27]
One experimental EVOO from Italy	n.i.	**Time:** 13 months **Conditions:** dark and light (illumination 10–12 h/day), type: n.i.; 20–22 °C **Type of container:** PET containing an oxygen scavenger; simple PET; 300 mL	Quality parameters; carotenes (spectrophotometrically); chlorophylls (spectrophotometrically); volatile compounds (HS-SPME-GC-MS)	[16]
39 commercial OOs from Spain	n.i.	**Time:** 9 months **Conditions:** non-specified **Type of container:** n.i.	Quality parameters; phenolic compounds (LC-DAD); volatile components (GC-MS); tocopherol, chlorophyll, carotenoid compounds and β-carotene (spectrophotometrically); oxidative stability (Rancimat); antioxidant capacity (DPPH); bitterness index (K_225_)	[5]
Four experimental EVOOs from Italy	*Nocellara Messinese* (2); *Piricuddara; Vaddarica*	**Time:** 18 months **Conditions:** darkness; RT **Type of container:** glass dark bottles; 1 L	Quality parameters; chlorophyll and carotenoid content (spectrophotometrically); phenolic compounds (LC-MS; quadrupole Orbitrap mass spectrometer)	[7]
Seven commercial EVOOs from Australia	*Arbequina; Barnea; Coratina; Frantoio; Koroneiki; Leccino; Picual*	**Time:** 24 months **Conditions:** light (type: n.i.); 20 and 30 °C **Type of container:** dark glass, clear glass and dark plastic; volume non-informed	Quality parameters; total polyphenol content; bitterness index (K_225_); pyropheophytins a; 1,2-diacylglycerols	[28]
One experimental EVOOs from Italy	*Moraiolo*	**Time:** 1 year **Conditions:** daylight; 25 °C **Type of container:** greenish glass bottles (headspace air; N_2_; Ar); 100 mL	Quality parameters; total phenol (Folin-Ciocalteau); phenolic compounds (LC-MS/MS); antioxidant capacity (ABTS); bitterness index (K_225_); sensory analysis	[29]
One commercial EVOO from Algeria	*Chemlal*	**Time:** 12 months **Conditions:** 6, 25 and 45 °C (daylight) **Type of container:** PET; 1 L	Quality parameters	[30]
Three EVOOs experimental from Italy	n.i.	**Time:** 24 months **Conditions:** RT **Type of container:** tin can and bag-in-box 1.5 and 3.0 L	Quality parameters; total phenol (Folin-Ciocalteau); phenolic compounds (LC-DAD); oxidative stability (Rancimat); sensory attributes	[31]
Three commercial EVOOs from Spain	*Picual; Arbequina; Picudo*	**Time:** 12 months **Conditions:** 12 h day ambient light and dark; RT **Type of container:** clear glass, dark glass, and PET	Quality parameters; phenolic compounds (IOOC method); phenolic compounds (UPLC-MS); sensory analysis	[32]
Seven experimental EVOOs from Slovenia	*Istrska* *belica* (4); *Leccino* (3)	**Time:** 142 h **Conditions:** 100 °C with air flow 10 L h^−1^ (Rancimat study)	Quality parameters; tocopherol (LC-FLD); total phenolic compounds (Folin-Ciocalteau); phenolic composition (LC-DAD)	[18]
One experimental EVOO from Argentine	*Arauco*	**Time:** 76 days **Conditions:** 24 h light (type: LED) and dark; 24 and 40 °C **Type of container:** dark glass bottles and PET (headspace air and N_2_); 150 mL	Quality parameters; total phenols after acid hydrolysis (LC-DAD); phenolic and pentacyclic triterpenic compounds (LC-MS)	Current work

Abbreviations used: n.i.: not informed; PET: polyethylene; RT: room temperature.

**Figure 1:**
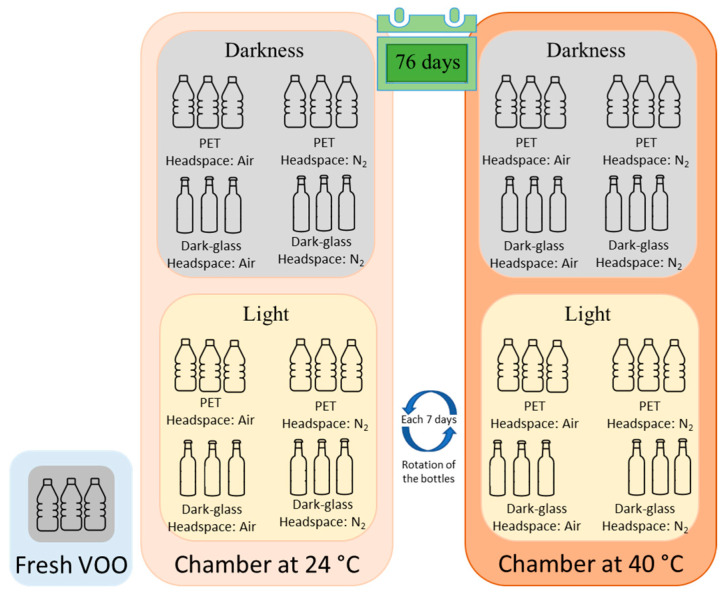
Outline of the study presented herein, describing the different tested storage conditions of *Arauco* VOO.

In the current research, the changes in the physicochemical parameters of *Arauco* VOO samples after storage under different conditions for 76 days were investigated. This experimental work was carried out to study the influence that exposure to light (darkness and light), temperature (24 and 40 °C), packaging material (polyethylene (PET) and dark glass), and headspace (air and N_2_ atmosphere) have on the quality of *Arauco* VOO (the complete experimental design can be seen in Figure 1). After storage, the basic quality parameters such as free acidity, peroxide values (PV), extinction coefficients, and fatty acid composition were measured. In addition, a liquid–liquid extraction followed by an LC-ESI-MS methodology was used to determine the profile of phenolic and pentacyclic triterpenoids. Phenolic compounds were also determined by acid hydrolysis followed by LC-DAD, to obtain the concentration of tyrosol (TY) and HTY derivatives. The latter methodology was applied to assess whether the oils, after storage, fulfilled the requirement of the “healthy food” claim established by the European Food Safety Authority (EFSA; at least 5 mg of HTY and its derivatives per 20 g of oil) [24]. The importance of the present study lies in the fact that it will provide information on the changes produced by different storage conditions in the composition of oils from this particular olive variety (for which there is no information on storage stability). As mentioned above, the composition of olive oil depends on numerous variables, among which the variety used is of vital importance. This work applied an experimental design that encompassed a large number of variables and the determination of many physicochemical parameters and compounds (many of them not measured in other works of this type); it will undoubtedly lead to valuable outcomes in the field of the storage stability of olive oils.

## 2. Results and Discussion

### 2.1. Quality Parameters

The quality parameters of fresh *Arauco* VOO (without storage treatment) were determined. The values of free acidity, PV, specific extinction coefficients (K_232_, K_268_, and ∆K), and fatty acid composition are summarized in Table 2. As can be seen, all values were below the limits set by International Olive Council (IOC) for extra VOO (EVOO) [33]. In order to study the influence of temperature on oil preservation, as stated before, *Arauco* VOO was exposed to two different temperatures, 24 and 40 °C, which could be reached during storage or transport without temperature control. These two temperatures could lead to different degradation kinetics. Similar temperatures have been used in other degradation studies related to the storage and transport of edible oils [9]. In addition, the photooxidation process was studied by applying light (570 lx), an intensity typically found in supermarkets where LED light is used. These temperature and light conditions were combined, as explained in Materials and Methods, with the use of two different types of containers and headspace with air or N_2_. Please note that from now on, the code that includes information about temperature/darkness or light (D or L)/type of container/N_2_ or air of the treatment will be used to identify the samples.

**Figure 2:**
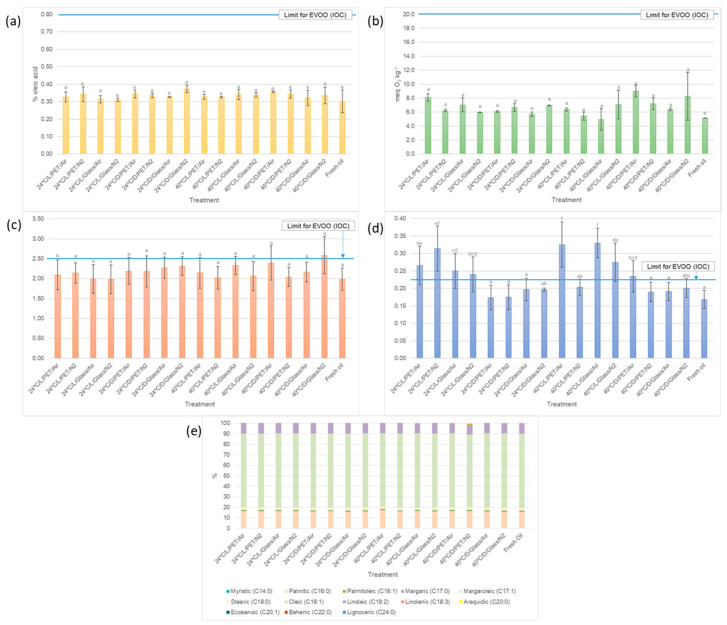
Results of the physicochemical characterization of the *Arauco* VOO samples (fresh and after applying the different storage conditions): (**a**) free acidity, (**b**) PV, (**c**) spectrophotometric index at 232 (K_232_), (**d**) spectrophotometric index at 268 (K_268_), and (**e**) fatty acid composition. The codes used on the *x*-axis include information for each treatment on temperature/darkness or light (D or L)/container type/N_2_ or air. The data are the mean and standard deviation of three independent experimentations. Different letters indicate significant differences among treatments at *p ≤* 0.05. The horizontal blue line (**a**–**d**) represents the IOC limit for EVOO [33].

After the treatments, VOO samples were analyzed to establish their main physicochemical characteristics, i.e., free acidity, PV, spectrophotometric indices (K_232_, K_268_, and ∆K), and fatty acid profile. Table S1 shows VOO’s quality parameters after storage in the different types of containers and tested conditions. For a better interpretation of the results, Figure 2a–d show the obtained values for free acidity, PV, and spectrophotometric indices (K_268_ and K_232_) after applying the different storage conditions over 76 days. In the aforementioned graphs, the last bar corresponds to fresh *Arauco* oil. With regard to free acidity levels and PV (Figure 2a,b), all samples retained the category of EVOO according to the IOC standard [33]. In particular, the fresh VOO sample (which presented the lowest value) and those from the different treatments did not show statistically significant differences in free acidity. The free acidity level increased from the initial value of 0.30 to a maximum value of 0.37%, which was still significantly below the limit of 0.80%. This behavior has been observed by other authors in samples with a high content of antioxidant compounds [16,34]. Some authors have stated in interesting contributions that as a result of hydrolytic degradation of triglycerides, the free acidity in oil may increase with storage at high temperature [34]. Figure 2b also shows that at 24 °C, the PVs were lower than at 40 °C in general, reaching the highest average value in the 40 °C/D/PET/air treatment. It was detected that at 24 °C in the presence of light, the PVs were slightly higher than in darkness. This could be explained considering the antioxidant power of chlorophylls in the dark. It should also be taken into account that the *Arauco* variety is a late harvest variety (this variety remains green for a long time, making it difficult to harvest with a high ripening index) [25]. At 40 °C, the highest PV values were reached in darkness, a fact that had been previously described by other authors [16,35]. The use of N_2_ as headspace had no clear influence on PV values. At 24 °C, the packaging material that seemed to preserve the oil better was glass; however, in the dark, the values were almost equal when comparing PET vs. glass. At 40 °C, the packaging material did not seem to have a consistent behavior. Possibly, the higher heat transfer of glass, together with other variables, generated this somewhat unspecific behavior.

Spectrophotometric indices, which are known to provide information on the oxidative state of the oil, exceeded, in some cases, the limit of an EVOO, as can be seen in Figure 2c,d. K_232_, used as an index of recent oxidation, did not show statistically significant differences between the oils from the different treatments and the fresh oil. However, the sample subjected to the 40°C/D/Glass/N_2_ treatment slightly exceeded the maximum limit of EVOO (Figure 2c), reaching a value of 2.58 (maximum limit 2.50). Comparing the light conditions, it is possible to observe that the oils stored at 24 °C and exposed to light had slightly lower K_232_ values than those stored in darkness. Under exposure to light and at 24 °C, the glass seemed to preserve better than the PET container, but in darkness, an inverse tendency was observed. At 40 °C, no clear trends were observed that allowed the comparison of the effects of the treatments. The results of K_268_, related to secondary oxidation, can be seen in Figure 2d (and Table S1). At the end of the study, some treatments resulted in oils whose values were above the legal limit. In addition, some treated oils (24 °C/L/PET/Air, 24 °C/L/PET/N_2_, 24 °C/L/Glass/Air, 24 °C/L/Glass/N_2_, 40 °C/L/PET/Air, 40 °C/L/Glass/Air, 40 °C/L/Glass/N_2_, and 40 °C/D/PET/Air) showed significant differences with the fresh starting oil. The evolution of this parameter at the two studied temperatures was quite similar, showing higher values in samples stored in the presence of light. It seems pertinent to point out again that *Arauco*, as a late variety, is rich in chlorophylls and that these compounds, in the absence of light, may inhibit the initiation stage of auto-oxidation processes. Similar behaviors have been previously documented for late varieties such as *Sikitita* [2,34,35]. The use of N_2_ as headspace, in most cases, seems to avoid to some extent the generation of oxidizing species.

In the present study and trying to establish some generalities with respect to what is observed in Figure 2a–d, lighting conditions appeared to have a greater influence on physicochemical parameters than thermal conditions or packaging material. When comparing the same treatments in terms of packaging material, temperature, and headspace, it was observed that the tendency for the quality parameters was that the values in the dark studies were higher (although not statistically significantly different) than those in the light treatments (except for the parameter K_268_).

The fatty acid composition of *Arauco* VOO before and after treatments is given in Table S1 and Figure 2e. No notable differences were found among the samples subjected to particular treatments, but there was a slight variation on the fatty acid composition of the samples after storage with respect to the fresh *Arauco* VOO. A slight decrease in oleic and linolenic acids (unsaturated) was observed, without significant differences between samples. These results are in agreement with the previous works reported [7]. As other authors have explained, the degradation of fatty acids was a consequence of their oxidation, so the rate of fatty acid degradation increased with the number of double bonds [3,7,18]. However, for oils with relatively high concentrations of phenolic compounds, the change in fatty acids was very limited during storage, remaining practically in the same proportion [18].

### 2.2. Determination of Total Content of HTY and TY-Related Compounds

**Figure 3:**
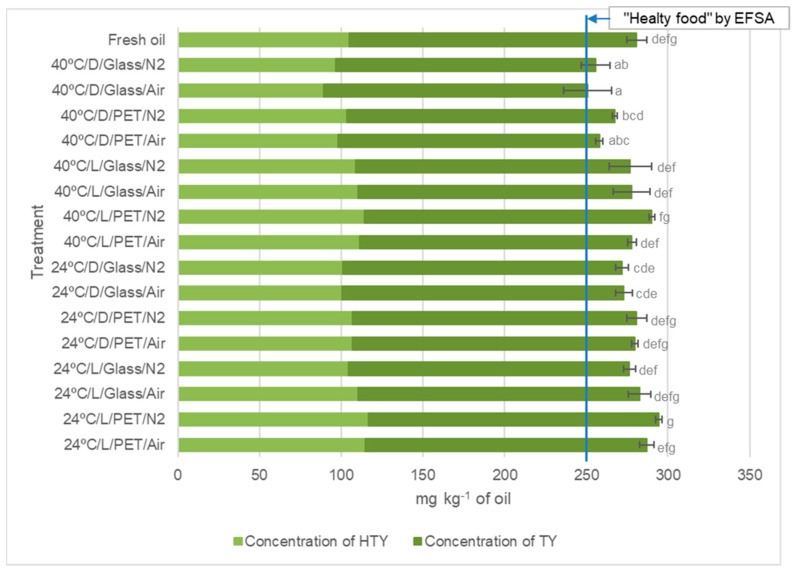
After-hydrolysis HTY and TY concentrations (mg kg^−1^ of oil) found in the *Arauco* VOO samples (fresh and after applying the different storage conditions). Different letters indicate significant differences among treatments at *p ≤* 0.05.

As noted above, phenolic compounds can be determined to monitor the changes that the oil undergoes during storage at different conditions and to assess whether *Arauco* VOO can be considered as a healthy food according to the EFSA declaration [36]. The acid hydrolysis results (HTY and TY content) obtained in the oils subjected to different treatments are summarized in Figure 3 (and Table S2). All treatments, even the most aggressive, led to oils complying with the EFSA claim, maintaining the health food status. A significant number of treatments resulted in oils that did not show statistically significant differences with respect to fresh oil. The oils with the lowest phenol concentration (measured as the sum of HTY and TY after hydrolysis) were those from the 40 °C/D/Glass/Air, 40 °C/D/Glass/N_2_, and 40 °C/D/PET/N_2_ treatments (which showed statistically significant differences with respect to the fresh oil); however, they still maintained a concentration above 250 mg of HTY and TY derivatives per kg^−1^ of oil. Overall, the results revealed that the preservation of the oils in PET seemed adequate (slightly better than glass, although this was not possible to state with certainty with this sample set), with improved stability when N_2_ was used in the headspace and at low temperature. It was difficult to compare the results achieved here with other previously published results because the methodologies for the determination of phenolic compounds (e.g., Folin-Ciocalteu) or the experimental design might be different (Table 1).

### 2.3. Bioactive Compounds Profiling

**Figure 4:**
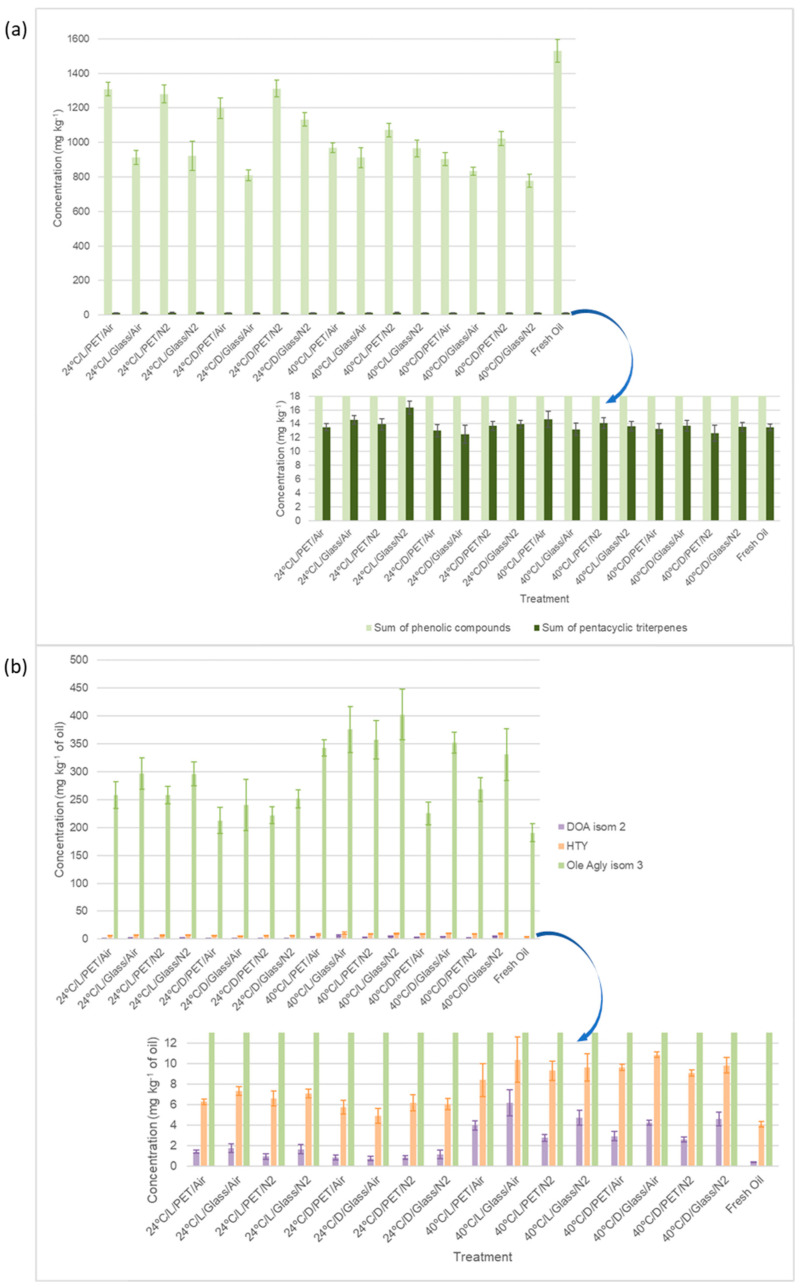
(**a**) Bar chart representing the sum of quantified bioactive compounds after each treatment, separated into phenolic compounds and pentacyclic triterpenes (mg kg^−1^ of oil). (**b**) Bar chart showing the concentration (mg kg^−1^ of oil), after each treatment, of the different compounds that could be considered as potential aging markers of *Arauco* VOO (DOA: decarboxymethyl oleuropein aglycone; isom: isomer; HTY: hydroxytyrosol; Ole Agly: oleuropein aglycone).

The profiling of bioactive compounds was determined to explore the evolution of these substances with the different treatments and their possible involvement in delaying or inhibiting oxidative phenomena in *Arauco* VOO. The methodology used was previously developed for a multi-class analysis [37], so the quantification of 31 compounds was possible, including phenolic compounds and pentacyclic triterpenes. The results of the quantitative assessment of the phenolic and triterpenic compounds in all the studied samples are shown in Table S3; as can be seen, the profile was strongly dominated by secoiridoids. Figure 4a displays the total content of the mentioned chemical families, expressed as the sum of 28 and three compounds, respectively, for phenols and triterpenes. All treatments showed gradual decreases in the summation concentration of fresh *Arauco* oil after the storage time. The 24 °C/D/PET/N_2_ treatment produced the slightest decrease in the total content of bioactive compounds, followed closely by the 24 °C/L/PET/Air, 24 °C/L/PET/N_2_, and 24 °C/D/PET/Air treatments. The observed loss percentages ranged from 14% to 49%, with the highest rate of loss occurring at 40 °C/D/Glass/N_2_. As far as pentacyclic triterpenes are concerned, the diverse treatments did not lead to oils that exhibited statistically significant differences among their results. This behavior could be explained by the high stability of this family of compounds, which are generally not greatly affected by the temperatures used [38], the light conditions applied, or the packaging materials explored in this study.

When analyzing the phenolic profiles, these were clearly dominated by secoiridoid derivatives, where ligstroside and oleuropein aglycones were prevalent, the latter being the one with the highest level found. Among the minor phenolic compounds studied, such as phenolic acids, flavonoids, or lignans, there was no noticeable change after the different treatments; a slight reduction in concentration could be observed after some treatments but without a pronounced trend.

It must be noticed that phenolic compounds are important natural antioxidants that are involved in several reactions. For example, they can chelate metals ions, inhibit lipid oxidation, or scavenge molecular species of active oxygen [3,4]. Therefore, it is of utmost interest to study the evolution of the different phenolic compounds after applying various storage conditions in order to verify the effects and influence of these compounds on the shelf life of the VOO. It has been published that some phenolic compounds, such as HTY and decarboxymethyl oleuropein aglycone isomer 2 (named as oleocanthalic acid by some authors [11,17]), could be used as ageing indicators, i.e., it has been observed that these compounds increase substantially in aged VOO [11]. For this reason, in the present study, special attention was paid to these ageing markers and to the changes that their concentrations might undergo after storing the oil at different conditions for 76 days. As can be deduced from Figure 4b, these compounds followed the trend described by other authors for VOO of different varieties and other storage conditions. The present investigation also suggested that these compounds could be used as ageing indicators for *Arauco* oils. This finding is particularly noteworthy as there is a need to find reliable markers that can be successfully measured and used for VOO samples of different varietals and geographical locations, from different extraction methods, etc.

Exploring in detail the evolution of certain secoiridoids, some interesting behaviors have been observed. Table S3 shows that three isomers of the oleuropein aglycone (Rt: 10.0, 12.6, and 13.2 min, respectively) were quantified. Two of them, Rt: 10.0 and 12.6 min, declined in concentration (compared with fresh VOO) after applying the different treatments. The degradation of these compounds was more severe at 40 °C than at 24 °C, while at 40 °C, the headspace had no influence on the stability of these substances. Light and packaging did have an influence, where PET proved to be better than glass. However, the third of the oleuropein aglycone isomers (Rt: 13.2 min) systematically increased in terms of concentration after the different storage processes (Figure 4b). The increase in this compound and its clear correlation with the intensity of the treatments led to the hypothesis that it is possible that this oleuropein derivative could be used as another indicator of VOO ageing. Specific studies are needed to confirm this, but it is undoubtedly a very promising discovery.

## 3. Materials and Methods

### 3.1. Samples

VOO from *Arauco* olives harvested in 2021 (the 2020–2021 Argentina season) was extracted using a two-phase system. The oil extraction was performed in cold without adding water at any stage of the process. The oil was filtered through a cotton layer and then transferred to dark glass bottles and stored in darkness at 4 °C.

### 3.2. Storage Treatments

For this study, *Arauco* VOO was packaged in different types of containers that were all filled to the top (the headspace in each bottle was about 1 mL); specifically, the containers used were PET bottles (150 mL) and dark glass bottles (150 mL). The bottles were sealed, and to evaluate the effect of the headspace of the vessel, a final step was performed before sealing half of the bottles in order to completely replace the air in the headspace with N_2_.

The containers were exposed to two temperatures (24 and 40 °C) and two light conditions (darkness and light (LED, 570 lx)) for a total of 76 days. The dark storage bottles were placed in a cardboard box in the same light chamber as the light treatment (same temperature and general conditions). The study was performed in triplicate (three independent experiments for each storage condition), and the containers were positionally rotated every seven days. The complete experimental design of this study can be seen in Figure 1.

### 3.3. Determination of Quality Indices

Free acidity, PV, spectroscopic indices (K_232_, K_268_, and ∆K), and fatty acid analyses were carried out according to the methods described by International Olive Council (IOC) standard methods, COI/T.15/NC Nº 3/Rev. 19 [33].

### 3.4. Total Content of HTY and TY Derivatives: Acid Hydrolysis of Secoiridoids

Secoiridoids hydrolysis was carried out following the protocol reported by Romero and Brenes with slight modifications [14]. Briefly, 0.5 g (±0.01 g) of VOO and 5 mL of HCl (2 M) were mixed in an orbital shaker at 400 rpm and room temperature for 6 h. Afterward, the aqueous phase was separated, filtered through a 0.22 µm nylon syringe filter, and finally analyzed by using an Agilent 1260 LC system (Agilent Technologies, Waldbronn, Germany), with DAD detection at 280 nm. A Zorbax Eclipse Plus C_18_ column (4.6 × 150 mm, 1.8 µm particle size), operating at room temperature, was used to separate the analytes of interest, applying the solvent gradient reported by Bajoub et al. [39]. Extracts from each of the three independent replicates were injected twice. HTY and TY calibration curves were prepared in HCl 2 M within a concentration range from 0.5 to 100 mg L^−1^. The injection volume was 10 µL for both extracts and standards.

### 3.5. LC-MS Profiling of Phenolic and Triterpenic Compounds

The individual quantification of VOO bioactive compounds was performed using the methodology previously reported by Olmo-García et al. [15]. Briefly, 1 g (±0.01 g) of VOO was extracted three successive times (vortex shaking, centrifugation, and supernatant collection): once with 10 mL of ethanol:water (60:40, *v*/*v*) and twice with 10 mL of ethanol:water (80:20, *v*/*v*). The extracts were combined, and the solvent was evaporated to dryness under reduced pressure at 35 °C. The obtained residue was reconstituted in 1 mL of ethanol:water (80:20, *v*/*v*) and filtered through a 0.22 µm nylon syringe filter.

The LC-MS analyses were conducted on an Agilent 1260 LC system (Agilent Technologies) coupled to a Bruker Daltonics Esquire 2000^TM^ ion trap mass spectrometer (Bruker Daltonik, Bremen, Germany) by means of an electrospray ionization source. A volume of 10 µL of the extracts and pure standards solutions was injected into the system, and the analytes were separated in a Zorbax Extend C_18_ column (4.6 × 100 mm, 1.8 µm particle size) at 40 °C with acidified water and acetonitrile (both with 1% acetic acid) as mobile phases at a flow rate of 1 mL min^−1^. Extracts from each of the three independent replicates of each tested storage condition were injected twice. The mobile phase gradient employed, as well as MS detection conditions, were comprehensively detailed elsewhere [37]. The MS spectra were acquired in negative ion mode within an m/z range from 50 to 1000. The following source parameters were adopted for IT MS: capillary voltage, +3200 V; drying gas (N_2_) flow and temperature, 9 L min^−1^ and 300 °C, respectively; nebulizer pressure, 30 psi. The quantification of individual compounds was performed by external calibration with standards solutions; each compound was quantified in terms of its own standard or the most similar molecule (if the pure standard was not commercially available or accessible in our laboratory). Secoiridoid derivatives, for instance, were quantified using the calibration curve of oleuropein.

### 3.6. Statistical Analysis

Analysis of variance followed by a Tukey test was performed on the acquired data with InfoStat statistical software (InfoStat version 2022, Grupo InfoStat, Córdoba, Argentina). The analysis of the variance indicated significant differences between the treatments and the treatment–time interaction (*p* ≤ 0.05).

## 4. Conclusions

The present study assessed the evolution during storage (at different conditions) for 76 days of Arauco—the only Argentinian variety—oil. For free acidity and PV values, all the samples remained within the limits established by the IOC and very far from the maximum value established for an EVOO, with no significant differences between the oils coming from the different treatments and the fresh sample. The K indices showed that the oil lost its EVOO quality only under extreme conditions, remaining as VOO. The fatty acid composition showed no significant differences for the oils from the different treatments. The total phenols after acid hydrolysis were significantly reduced after applying some of the storage conditions and were not affected by others; in any case, all samples still complied with the EFSA declaration after any of the treatments. Overall, the results revealed that the PET container appeared to be adequate, with improved stability when N_2_ was used in the headspace, together with darkness and low temperature. It was also observed that the oils showed better resistance to light than to temperature. The study of the evolution of phenolic compound profiles showed that substances such as HTY, decarboxymethyl oleuropein aglycone isomer 2, and another compound derived from oleuropein (oleuropein aglycone isomer 3) exhibited consistent behavior as Arauco oil ages; hence, they could be used as ageing markers.

## Figures and Tables

**Table 2:** Physicochemical characterization of fresh *Arauco* EVOO.

Analytical Parameters		Fresh *Arauco* Oil ^&^	Limit for EVOO *
Free fatty acids (% oleic acid)		0.30 ± 0.07	≤0.80
PV (meq O_2_ kg^−1^)		5.13 ± 0.02	≤20.0
K_232_		0.17 ± 0.02	≤2.50
K_268_		2.14 ± 0.06	≤0.22
∆K		0.00 ± 0.00	≤0.01
Fatty acids:			
	Myristic acid (C14:0)	0.01 ± 0.00	≤0.03%
	Palmitic acid (C16:0)	15.71 ± 0.28	7.00–20.00%
	Palmitoleic acid (C16:1)	1.08 ± 0.02	0.30–3.50%
	Margaric acid (C17:0)	0.04 ± 0.00	≤0.40%
	Margaroleic acid (C17:1)	0.05 ± 0.00	≤0.60%
	Stearic acid (C18:0)	2.52 ± 0.08	0.50–5.00%
	Oleic acid (C18:1)	70.83 ± 0.06	55.00–85.00%
	Linoleic acid (C18:2)	8.85 ± 0.26	2.50–21.00%
	Linolenic acid (C18:3)	0.45 ± 0.01	≤1.00%
	Araquidic acid (C20:0)	0.23 ± 0.00	≤0.60%
	Eicosenoic acid (C20:1)	0.13 ± 0.00	≤0.50%
	Behenic acid (C22:0)	0.02 ± 0.01	≤0.20%
	Lignoceric acid (C24:0)	0.08 ± 0.00	≤0.20%

^&^ Every result is expressed as the average ± the standard deviation of three independent replicates. * Limits stablished for EVOO according to the IOC [33].

## Data Availability

The data presented in this study are available in Table 2, Figure 2, Figure 3 and Figure 4, and all the Supplementary Materials.

## Supplementary Materials

The following supporting information can be downloaded at: https://www.mdpi.com/article/10.3390/plants12091826/s1, Table S1: Physicochemical parameters of the *Arauco* VOO samples after applying the different storage conditions. Table S2: Concentrations of HTY and TY after acid hydrolysis found in the *Arauco* VOO samples by LC-DAD. Table S3: Quantitative results obtained for the *Arauco* VOO samples by LC-ESI-IT MS.
