# Supplementary material for: Storage Stability of Arauco Virgin Olive Oil: Evolution of Its Quality Parameters and Phenolic and Triterpenic Compounds under Different Conservation Conditions
Source: Plants (Basel). 2023 Apr 29;12(9):1826. doi: 10.3390/plants12091826 (PMC10181468; doi:10.3390/plants12091826)
Supplement: Supplementary file 1 [file plants-12-01826-s001.zip › plants-2346614-supplementary.pdf]

Article

Storage Stability of *Arauco* Virgin Olive Oil: Evolution of Its Quality Parameters and Phenolic and Triterpenic Compounds under Different Conservation Conditions

Romina P. Monasterio [1,2,*], Eduardo Trentacoste [3], Carlos López Appiolaza [1], María Gemma Beiro-Valenzuela [2], Irene Serrano-García [2], Lucía Olmo-García [2] and Alegría Carrasco-Pancorbo [2]

Table S1: Physicochemical parameters of the *Arauco* VOO samples after applying the different storage conditions.

Analytical parameters	Limit for EVOO*	24ºC/L/ PET/Air&	24ºC/L/ PET/N_2&	24ºC/L/ Glass/Air&	24ºC/L/ Glass/N_2&	24ºC/D/ PET/Air&	24ºC/D/ PET/N_2&	24ºC/D/ Glass/Air&	24ºC/D/ Glass/N_2&
Free fatty acids (% oleic acid)	**≤0.80**	0.33±0.03	0.34±0.04	0.31±0.02	0.31±0.01	0.34±0.02	0.33±0.01	0.33±0.00	0.37±0.02
Peroxide value (meq O_2 kg^{-1})	**≤20.0**	8.09±0.52	6.23±0.17	7.04±1.00	5.97±0.05	6.06±0.11	6.67±0.56	5.72±0.32	6.96±0.04
K_{232}	**≤2.50**	2.09±0.38	2.14±0.25	1.99±0.36	1.98±0.36	2.19±0.34	2.18±0.39	2.27±0.27	2.31±0.23
K_{268}	**≤0.22**	0.27±0.06	0.31±0.07	0.25±0.05	0.24±0.05	0.17±0.04	0.18±0.04	0.20±0.03	0.20±0.00
ΔK	**≤0.01**	0.00	0.00	0.00	0.00	0.00	0.00	0.00	0.00
Fatty acids:									
Myristic acid (C14:0)	**≤0.03%**	0.01±0.00	0.01±0.00	0.01±0.00	0.01±0.00	0.01±0.00	0.01±0.00	0.01±0.00	0.01±0.00
Palmitic acid (C16:0)	**7.00-20.00%**	16.33±0.07	16.29±0.27	16.38±0.41	16.36±0.41	15.99±0.30	16.23±0.09	15.87±0.08	16.11±0.42
Palmitoleic acid (C16:1)	**0.30-3.50%**	1.16±0.00	1.16±0.03	1.15±0.05	1.16±0.06	1.13±0.02	1.14±0.01	1.10±0.01	1.15±0.09
Margaric acid (C17:0)	**≤0.40%**	0.04±0.00	0.04±0.00	0.04±0.00	0.04±0.00	0.04±0.00	0.04±0.00	0.04±0.00	0.05±0.01
Margaroleic acid (C17:1)	**≤0.60%**	0.05±0.00	0.05±0.00	0.06±0.01	0.05±0.01	0.05±0.00	0.05±0.00	0.05±0.00	0.05±0.01
Stearic acid (C18:0)	**0.50-5.00%**	2.42±0.01	2.43±0.03	2.44±0.03	2.43±0.03	2.46±0.03	2.46±0.03	2.49±0.03	2.45±0.03
Oleic acid (C18:1)	**55.00-83.00%**	70.29±0.07	70.23±0.29	70.37±0.31	70.45±0.15	70.42±0.27	70.27±0.06	70.70±0.12	70.63±0.27
Linoleic acid (C18:2)	**2.50-21.00%**	8.87±0.03	8.95±0.06	8.75±0.15	8.68±0.23	9.05±0.00	8.95±0.00	8.88±0.16	8.68±0.17

Linolenic acid (C18:3)	**≤1.00%**	0.39±0.02	0.41±0.01	0.40±0.01	0.39±0.04	0.42±0.02	0.43±0.01	0.42±0.02	0.41±0.04
Araquidic acid (C20:0)	**≤0.60%**	0.23±0.01	0.22±0.01	0.22±0.02	0.23±0.02	0.21±0.01	0.22±0.01	0.22±0.01	0.23±0.01
Eicosenoic acid (C20:1)	**≤0.50%**	0.12±0.01	0.11±0.00	0.12±0.01	0.12±0.02	0.12±0.01	0.12±0.01	0.12±0.00	0.12±0.00
Behenic acid (C22:0)	**≤0.20%**	0.02±0.00	0.02±0.00	0.02±0.00	0.02±0.00	0.02±0.00	0.02±0.00	0.02±0.01	0.02±0.00
Lignoceric acid (C24:0)	**≤0.20%**	0.07±0.00	0.07±0.01	0.06±0.01	0.06±0.02	0.07±0.00	0.07±0.00	0.07±0.00	0.07±0.01

Table S1: Continued.

Analytical parameters	40ºC/L/ PET/Air[&]	40ºC/L/ PET/N_2[&]	40ºC/L/ Glass/Air[&]	40ºC/L/ Glass/N_2[&]	40ºC/D/ PET/Air[&]	40ºC/D/ PET/N_2[&]	40ºC/D/ Glass/Air[&]	40ºC/D/ Glass/N_2[&]	Fresh oil[&]
Free fatty acids (% oleic acid)	0.33±0.01	0.33±0.00	0.34±0.03	0.34±0.01	0.36±0.00	0.34±0.02	0.32±0.04	0.34±0.05	0.30±0.07
Peroxide value (meq O_2 kg^{-1})	6.38±0.24	5.43±0.58	4.96±1.55	7.09±2.05	9.02±0.84	7.20±0.84	6.38±0.20	8.28±3.43	5.13±0.02
K_{232}	2.15±0.39	2.02±0.28	2.33±0.22	2.06±0.37	2.39±0.43	2.04±0.24	2.16±0.25	2.58±0.47	1.98±0.28
K_{268}	0.33±0.07	0.20±0.02	0.33±0.04	0.27±0.06	0.23±0.05	0.19±0.03	0.19±0.03	0.20±0.03	0.17±0.02
ΔK	0.01±0.00	0.00	0.00	0.00	0.00	0.00	0.01±0.01	0.00	0.00
Fatty acids:									
Myristic acid (C14:0)	0.00±0.00	0.01±0.00	0.01±0.00	0.01±0.00	0.01±0.00	0.01±0.00	0.01±0.00	0.01±0.00	0.01±0.00
Palmitic acid (C16:0)	17.14±0.87	15.95±0.25	16.33±0.42	16.18±0.49	16.33±0.16	16.47±0.31	16.11±0.19	15.92±0.27	15.71±0.28
Palmitoleic acid (C16:1)	1.29±0.11	1.10±0.01	1.15±0.07	1.14±0.07	1.19±0.02	1.19±0.05	1.13±0.02	1.10±0.04	1.08±0.02
Margaric acid (C17:0)	0.04±0.00	0.04±0.00	0.04±0.00	0.04±0.00	0.04±0.00	0.04±0.00	0.04±0.00	0.05±0.01	0.04±0.00
Margaroleic acid (C17:1)	0.06±0.01	0.05±0.00	0.05±0.00	0.05±0.00	0.06±0.00	0.06±0.00	0.05±0.01	0.05±0.00	0.05±0.00
Stearic acid (C18:0)	2.38±0.03	2.50±0.01	2.45±0.04	2.48±0.04	2.40±0.01	2.40±0.03	2.45±0.02	2.49±0.07	2.52±0.08
Oleic acid (C18:1)	69.52±0.91	70.58±0.19	70.47±0.33	70.45±0.28	70.36±0.14	69.22±1.05	70.76±0.20	70.78±0.17	70.83±0.06
Linoleic acid (C18:2)	8.80±0.03	8.94±0.05	8.64±0.08	8.82±0.24	8.77±0.02	8.87±0.13	8.58±0.01	8.73±0.10	8.85±0.26
Linolenic acid (C18:3)	0.36±0.04	0.42±0.00	0.41±0.02	0.42±0.01	0.40±0.00	0.39±0.02	0.40±0.03	0.42±0.04	0.45±0.01
Araquidic acid (C20:0)	0.20±0.02	0.21±0.00	0.22±0.02	0.22±0.00	0.22±0.00	0.23±0.00	0.24±0.01	0.22±0.01	0.23±0.00
Eicosenoic acid (C20:1)	0.12±0.01	0.12±0.01	0.13±0.00	0.12±0.00	0.12±0.01	0.12±0.01	0.13±0.01	0.13±0.02	0.13±0.00
Behenic acid (C22:0)	0.02±0.01	0.02±0.00	0.02±0.00	0.02±0.00	0.02±0.00	0.04±0.04	0.02±0.01	0.02±0.01	0.02±0.01
Lignoceric acid (C24:0)	0.06±0.02	0.07±0.00	0.09±0.01	0.06±0.01	0.07±0.01	0.07±0.01	0.07±0.01	0.08±0.01	0.08±0.00

*EVOO: limits stablished for EVOO according to the IOC [25].

[&]Every result is expressed as the average ± the standard deviation of three independent replicates.

Table S2: Concentrations of HTY and TY after acid hydrolysis (mg kg^{-1} of oil) found in the *Arauco* VOO samples (fresh and after applying the different storage conditions) by LC-DAD.

Treatment	HTY (mg kg^{-1})$^{\&}$	TY (mg kg^{-1})$^{\&}$	Sum HTY + TY (mg kg^{-1})*
24ºC/L/PET/Air	114.52±2.41	172.51±2.42	287.03±4.41^{efg}
24ºC/L/Glass/Air	110.35±2.96	172.26±3.85	282.61±6.71^{defg}
24ºC/L/PET/N_2	116.80±1.22	177.49±1.08	294.29±1.94^{g}
24ºC/L/Glass/N_2	112.25±2.29	170.51±12.00	282.76±13.15^{defg}
24ºC/D/PET/Air	106.95±1.99	172.76±0.39	279.71±2.17^{defg}
24ºC/D/Glass/Air	100.36±4.90	172.71±1.23	273.07±5.13^{cde}
24ºC/D/PET/N_2	106.69±3.12	174.18±4.04	280.88±6.27^{defg}
24ºC/D/Glas/N_2	100.87±3.97	170.94±0.34	271.80±4.07^{bcde}
40ºC/L/PET/Air	111.18±1.57	166.67±1.24	277.85±2.67^{defg}
40ºC/L/Glass/Air	110.24±5.00	167.61±6.45	277.85±11.36^{defg}
40ºC/L/PET/N_2	114.19±0.93	175.85±1.03	290.04±1.69f^{g}
40ºC/L/Glass/N_2	108.98±5.29	167.92±7.70	276.91±12.93^{def}
40ºC/D/PET/Air	97.98±2.48	160.15±1.20	258.12±2.23^{abc}
40ºC/D/Glass/Air	89.42±13.92	161.41±0.63	250.83±14.54^{a}
40ºC/D/PET/N_2	103.53±1.16	164.08±0.49	267.61±1.53^{bcd}
40ºC/D/Glass/N_2	96.78±4.14	159.02±4.85	255.80±8.76^{ab}
Fresh oil	104.93±4.89	175.89±2.21	280.82±6.23^{defg}

$^{\&}$The data is the mean, and standard deviation, of three independent experimentations.

*Means with a letter in common are not significantly different.

Table S3: Quantitative results (mg kg^{-1} of oil) obtained for the *Arauco* VOO samples (fresh and after applying the different storage conditions) by LC-ESI-IT MS.

Compound (Peak number)	Rt (min)	*m/z*	24ºC/L/ PET/Air$^{\&}$	24ºC/L/ Glass/Air$^{\&}$	24ºC/L/ PET/$N_2$$^{\&}$	24ºC/L/ Glass/$N_2$$^{\&}$	24ºC/D/ PET/Air$^{\&}$	24ºC/D/ Glass/Air$^{\&}$	24ºC/D/ PET/$N_2$$^{\&}$	24ºC/D/ Glass/$N_2$$^{\&}$	40ºC/L/ PET/Air$^{\&}$
Qui (1)	0.9	191	0.40±0.04^{a}	0.12±0.06^{a}	0.13±0.06^{a}	0.14±0.02^{a}	1.08±0.27^{a}	0.96±0.42^{a}	1.19±0.4^{a}	0.47±0.08^{a}	0.15±0.07^{a}
OxHTY (2)	0.9	151	0.39±0.04^{ab}	0.41±0.05^{ab}	0.40±0.07^{ab}	0.48±0.12^{ab}	0.39±0.05^{ab}	0.37±0.05^{a}	0.42±0.05^{ab}	0.42±0.05^{ab}	0.42±0.05^{ab}
HTY (3)	1.7	153	6.28±0.26^{bc}	7.31±0.43^{cde}	6.59±0.73^{bcd}	7.11±0.43^{cdef}	5.77±0.66^{abc}	4.91±0.74^{ab}	6.18±0.79^{bc}	6.05±0.55^{abc}	8.39±1.59^{defg}
TY (4)	2.6	137	11.37±1.00^{bcd}	10.38±2.88^{ab}	11.51±1.11^{bcd}	10.88±1.36^{abc}	9.76±1.11^{ab}	8.54±0.73^{a}	10.16±0.99^{ab}	9.77±0.85^{ab}	15.56±0.86^{efgh}
Van (5)	3.2	167	0.52±0.05	0.51±0.05	0.50±0.04	0.56±0.15	0.45±0.05	0.44±0.09	0.47±0.05	0.50±0.02	0.59±0.1
DEA (6)	4.2	183	0.07±0.01	0.07±0.01	0.06±0.01	0.08±0.01	0.06±0.01	0.06±0.01	0.07±0.01	0.06±0.01	0.06±0.01
p-Cou (7)	4.7	163	1.18±0.15^{ab}	1.18±0.12^{ab}	1.12±0.10^{ab}	1.10±0.08^{b}	1.13±0.12^{ab}	1.11±0.12^{ab}	1.21±0.11^{b}	1.22±0.09^{b}	1.00±0.05^{a}
Vanillin (8)	4.9	136	0.09±0.03	0.08±0.02	0.08±0.02	0.08±0.01	0.08±0.03	0.06±0.03	0.08±0.03	0.08±0.03	0.09±0.02
Fer (9)	5.8	193	0.23±0.03	0.24±0.04	0.23±0.04	0.25±0.06	0.22±0.04	0.16±0.09	0.23±0.04	0.24±0.03	0.21±0.03
DesoxyEA (10)	5.9	225	1.20±0.09	1.15±0.10	1.19±0.10	1.20±0.07	1.15±0.15	1.05±0.21	1.21±0.13	1.18±0.1	1.17±0.11
HEA (11)	7.0	257	0.07±0.01^{ab}	0.10±0.04^{abc}	0.07±0.01^{ab}	0.14±0.06^{bc}	0.05±0.01^{ab}	0.04±0.01^{a}	0.05±0.01^{ab}	0.06±0.01^{ab}	0.18±0.01^{cd}
EA (12)	8.0	241	6.77±1.20^{ab}	7.47±1.18^{abc}	6.53±1.32^{ab}	7.85±0.82^{abc}	5.38±1.65^{a}	4.65±1.03^{a}	6.39±1.58^{ab}	7.13±1.54^{ab}	6.41±0.84^{ab}
HTY AcyE (13)	8.9	381	0.05±0.01^{b}	0.06±0.01^{b}	0.05±0.00^{b}	0.05±0.01^{b}	0.05±0.01^{b}	0.06±0.01^{b}	0.06±0.01^{b}	0.06±0.01^{b}	0.02±0.01^{a}
HDOle Agly (14)	9.5	335	2.48±0.24^{bc}	2.90±0.80^{bc}	1.63±0.46^{ab}	3.65±1.21^{cd}	1.39±0.29^{ab}	1.09±0.14^{ab}	1.44±0.42^{ab}	1.80±0.38^{ab}	7.69±0.50^{gh}
Ole (15)	9.7	539	n.d.$^{\#}$	n.d.$^{\#}$	n.d.$^{\#}$	n.d.$^{\#}$	n.d.$^{\#}$	n.d.$^{\#}$	n.d.$^{\#}$	n.d.$^{\#}$	n.d.$^{\#}$
Ole Agly isom 1 (16)	10.0	377	235.26±14.77^{f}	52.90±6.24^{ab}	212.89±26.77^{f}	57.85±12.72^{ab}	230.57±31.44^{f}	90.90±7.42^{bcd}	253.69±21.74^{f}	153.06±17.84^{e}	68.46±9.34^{abc}
DOA isom 1 (17)	10.2	319	26.9±0.86	27.34±2.97	28.03±0.62	32.25±2.33	23.02±1.90	22.86±2.06	24.10±2.00	25.80±2.55	28.96±0.96
Lut (18)	11.4	285	3.42±0.12^{bc}	3.68±0.12^{bcd}	3.39±0.18^{b}	3.38±0.14^{b}	3.43±0.21^{bc}	2.95±0.38^{a}	3.52±0.17^{bcd}	3.40±0.17^{b}	3.93±0.11^{d}
DOA isom 2 (19)	12.1	319	1.41±0.15^{abc}	1.75±0.45^{bcd}	0.93±0.30^{ab}	1.65±0.47^{cde}	0.84±0.22^{ab}	0.74±0.19^{ab}	0.83±0.20^{ab}	1.13±0.44^{abc}	3.97±0.44^{fg}
Pin (20)	12.4	357	0.09±0.03^{ab}	0.13±0.05^{ab}	0.07±0.05^{a}	0.26±0.07^{c}	0.09±0.03^{ab}	0.12±0.04^{ab}	0.09±0.03^{a}	0.09±0.04^{ab}	0.13±0.05^{ab}
Ole Agly isom 2 (21)	12.6	377	246.27±19.18^{fg}	100.86±9.82^{bcd}	249.39±28.32^{fg}	107.18±21.57^{cd}	266.75±27.33^{fg}	231.35±41.01^{fg}	278.15±26.08^{g}	191.07±11.19^{ef}	29.75±3.13^{abc}
10-H Ole Agly (22)	12.7	393	12.16±3.45^{abc}	15.57±4.05^{bc}	7.64±3.23^{ab}	16.42±2.00^{bc}	5.57±1.77^{ab}	6.15±1.33^{ab}	6.16±2.07^{ab}	9.79±3.10^{ab}	48.94±2.45^{efg}
DLA (23)	12.7	303	3.49±0.24^{abc}	3.30±0.52^{abc}	3.67±0.28^{bcd}	3.21±0.16^{abc}	2.99±0.45^{ab}	3.05±0.58^{ab}	2.70±0.85^{ab}	3.46±0.50^{abc}	3.60±0.32^{bcd}
Api (24)	12.8	269	1.14±0.03	1.06±0.13	1.07±0.08	1.66±0.66	1.02±0.10	1.01±0.21	1.42±0.55	1.16±0.08	1.07±0.06
Dio (25)	12.9	299	0.28±0.04	0.22±0.02	0.25±0.03	0.33±0.13	0.23±0.04	0.18±0.07	0.26±0.04	0.24±0.03	0.26±0.04
Lig Agly Isom 1 (26)	13.2	361	312.08±9.09^{de}	211.47±24.06^{bc}	295.04±26.63^{cde}	217.00±22.91^{bc}	290.16±33.64^{cde}	188.02±12.11^{b}	323.06±25.87^{de}	295.73±24.43^{cde}	180.20±17.70^{b}
Ole Agly isom 3 (27)	13.2	377	257.89±23.85^{abc}	296.49±28.24^{cde}	258.07±15.33^{abc}	295.98±21.68^{cdef}	212.63±23.59^{ab}	240.28±24.82^{abc}	222.04±15.39^{ab}	251.34±15.89^{abc}	342.19±14.54^{efg}
Lig Agly Isom 2 (28)	13.7	361	177.87±14.54^{abcd}	165.75±12.41^{abc}	187.74±18.77^{bcde}	172.25±7.18^{abcd}	134.27±13.25^{ab}	95.74±6.65^{a}	166.20±17.72^{abcd}	168.52±13.44^{abcd}	217.00±13.55^{bcde}
MA (29)	15.7	471	8.69±0.21^{ab}	8.90±0.56^{ab}	8.84±0.52^{ab}	9.38±0.49^{b}	8.18±0.45^{ab}	7.85±0.75^{a}	8.43±0.57^{ab}	8.52±0.49^{b}	9.40±0.94^{ab}
BA (30)	17.4	455	0.07±0.01^{ab}	0.09±0.01^{ab}	0.06±0.03^{a}	0.09±0.01^{ab}	0.07±0.04^{ab}	0.05±0.01^{a}	0.08±0.03^{ab}	0.08±0.02^{ab}	0.10±0.02^{ab}
OA (31)	17.7	455	4.75±0.45^{ab}	5.56±0.28^{b}	5.04±0.61^{ab}	5.00±0.14^{ab}	4.74±0.80^{ab}	4.62±1.07^{ab}	5.18±0.39^{ab}	5.33±0.24^{ab}	5.12±0.67^{ab}

Table S3: Continued.

Compound	Rt (min)	m/z	40ºC/L/ Glass/Air[&]	40ºC/L/ PET/N_2[&]	40ºC/L/ Glass/N_2[&]	40ºC/D/ PET/Air[&]	40ºC/D/ Glass/Air[&]	40ºC/D/ PET/N_2[&]	40ºC/D/ Glass/N_2[&]	Fresh Oil[&]
Qui (1)	0.9	191	0.06±0.05^{a}	0.15±0.1^{a}	0.03±0.01^{a}	0.78±0.56^{a}	0.13±0.04^{a}	0.36±0.07^{a}	0.11±0.08^{a}	6.57±0.08^{b}
OxHTY (2)	0.9	151	0.50±0.09^{abc}	0.43±0.05^{ab}	0.54±0.1^{bc}	0.46±0.06^{ab}	0.73±0.10^{d}	0.46±0.04^{ab}	0.64±0.09^{cd}	0.38±0.04^{a}
HTY (3)	1.7	153	10.40±2.20^{gh}	9.31±0.64^{efgh}	9.63±1.36^{fgh}	9.65±0.29^{fgh}	10.87±0.29^{h}	9.08±0.29^{efgh}	9.83±0.75^{gh}	4.07±0.27^{a}
TY (4)	2.6	137	16.81±0.42^{h}	14.01±0.93^{defg}	17.09±0.68^{h}	13.60±0.51^{def}	16.45±0.38^{gh}	13.08±0.79^{cde}	16.25±0.73^{fgh}	8.37±0.71^{a}
Van (5)	3.2	167	0.54±0.09	0.50±0.09	0.54±0.04	0.47±0.09	0.49±0.11	0.54±0.08	0.52±0.08	0.52±0.07
DEA (6)	4.2	183	0.07±0.01	0.07±0.01	0.07±0.01	0.07±0.01	0.06±0.01	0.06±0.01	0.06±0.01	0.07±0.01
p-Cou (7)	4.7	163	1.14±0.08^{ab}	0.99±0.09^{ab}	1.07±0.07^{ab}	1.13±0.16^{ab}	1.04±0.19^{ab}	1.18±0.09^{ab}	0.93±0.10^{a}	1.17±0.10^{ab}
Vanillin (8)	4.9	136	0.07±0.04	0.08±0.01	0.08±0.02	0.07±0.02	0.10±0.01	0.07±0.02	0.09±0.02	0.08±0.04
Fer (9)	5.8	193	0.22±0.04	0.21±0.06	0.21±0.05	0.23±0.03	0.20±0.04	0.23±0.04	0.20±0.03	0.23±0.03
DesoxyEA (10)	5.9	225	1.16±0.1	1.17±0.09	1.13±0.07	1.14±0.09	1.13±0.07	1.16±0.06	1.10±0.04	0.85±0.06
HEA (11)	7.0	257	0.30±0.04^{e}	0.13±0.01^{abc}	0.30±0.10^{e}	0.13±0.01^{abc}	0.29±0.01^{e}	0.10±0.01^{abc}	0.26±0.08^{de}	0.04±0.01^{a}
EA (12)	8.0	241	10.68±1.34^{cd}	6.12±1.1^{a}	11.45±1.98^{d}	5.60±0.97^{a}	10.57±0.41^{cd}	5.49±1.14^{a}	9.58±1.43^{bcd}	7.11±1.38^{ab}
HTY AcyE (13)	8.9	381	0.02±0.01^{a}	0.02±0.01^{a}	0.02±0.01^{a}	0.02±0.01^{a}	0.02±0.01^{a}	0.02±0.01^{a}	0.02±0.01^{a}	0.19±0.02^{c}
HDOle Agly (14)	9.5	335	9.29±1.82^{h}	5.20±0.45^{de}	6.86±1.25^{efg}	6.89±0.57^{efg}	7.33±0.56^{fg}	5.60±0.52^{ef}	7.44±1.16^{g}	0.57±0.04^{a}
Ole (15)	9.7	539	n.d.[#]	n.d.[#]	n.d.[#]	n.d.[#]	n.d.[#]	n.d.[#]	n.d.[#]	0.32±0.03
Ole Agly isom 1 (16)	10.0	377	22.10±0.98^{a}	68.49±12.35^{ab}	25.59±5.71^{a}	122.52±8.18^{cde}	21.80±4.08^{a}	132.99±10.89^{de}	18.64±1.69^{a}	363.37±28.46^{g}
DOA isom 1 (17)	10.2	319	31.18±3.40	32.43±0.51	32.28±2.62	22±1.63	28.13±0.71	24.19±0.75	26.91±2.81	24.72±1.11
Lut (18)	11.4	285	3.67±0.10^{bcd}	3.49±0.18^{bc}	3.57±0.21^{bcd}	3.52±0.20^{bcd}	3.45±0.21^{bc}	3.72±0.10^{bcd}	3.53±0.20^{bcd}	3.84±0.08^{cd}
DOA isom 2 (19)	12.1	319	6.18±1.29^{h}	2.75±0.36^{de}	4.71±0.72^{g}	2.91±0.45^{ef}	4.22±0.24^{g}	2.60±0.24^{de}	4.61±0.65^{g}	0.36±0.04^{a}
Pin (20)	12.4	357	0.25±0.06^{c}	0.12±0.03^{ab}	0.27±0.07^{c}	0.09±0.03^{ab}	0.24±0.03^{c}	0.11±0.03^{ab}	0.20±0.05^{bc}	0.05±0.03a
Ole Agly isom 2 (21)	12.6	377	23.46±3.75^{ab}	58.08±6.67^{abcd}	22.73±1.37^{ab}	89.51±8.99^{abcd}	19.07±0.45^{a}	116.09±8.72^{de}	18.08±1.94^{a}	391.14±42.16^{h}
10-H Ole Agly (22)	12.7	393	54.68±6.93^{fg}	38.7±1.09^{e}	46.70±4.14^{efg}	42.66±1.76^{ef}	54.71±1.93^{fg}	38.10±1.88^{e}	51.50±6.67^{fg}	0.82±0.50^{a}
DLA (23)	12.7	303	4.98±1.49^{de}	4.52±0.29^{cde}	5.66±0.90^{e}	2.12±0.41^{a}	3.66±0.38^{bcd}	2.61±0.28^{ab}	3.36±0.66^{abc}	3.15±0.31^{abc}
Api (24)	12.8	269	0.97±0.14	1.19±0.04	1.10±0.10	1.03±0.07	0.97±0.07	1.17±0.10	1.01±0.07	1.05±0.12
Dio (25)	12.9	299	0.25±0.02	0.22±0.03	0.24±0.02	0.28±0.04	0.25±0.02	0.26±0.04	0.22±0.04	0.28±0.02
Lig Agly Isom 1 (26)	13.2	361	36.76±3.55+	215.84±9.83^{bc}	35.05±1.83^{a}	217.10±16.83^{bc}	35.05±1.83^{a}	229.84±32.28^{bcd}	33.38±4.54^{a}	354.84±28.73^{e}
Ole Agly isom 3 (27)	13.2	377	375.94±41.27^{fg}	357.08±34.3^{efg}	402.63±35.81^{g}	225.53±20.2^{ab}	351.98±19.03^{efg}	268.29±21.17^{bcd}	330.65±23.65^{def}	190.60±16.42^{a}
Lig Agly Isom 2 (28)	13.7	361	301.88±38.23^{fg}	250.47±9.68^{def}	337.49±32.36^{g}	133.94±24.45^{ab}	263.01±14.73^{efg}	164.73±20.98^{abc}	238.86±29.14^{cdef}	162.57±22.64^{abc}
MA (29)	15.7	471	8.48±0.40^{b}	9.28±0.50^{b}	8.97±0.47^{ab}	8.26±0.49^{ab}	8.52±0.40^{ab}	8.47±1.06^{ab}	8.43±0.46^{ab}	8.47±0.28^{ab}
BA (30)	17.4	455	0.06±0.03^{a}	0.06±0.01^{a}	0.09±0.03^{ab}	0.08±0.02^{ab}	0.12±0.04^{b}	0.08±0.01^{ab}	0.10±0.02^{ab}	0.10±0.02^{ab}
OA (31)	17.7	455	4.65±0.8^{ab}	4.77±0.63^{ab}	4.6±0.5^{ab}	4.93±0.56^{ab}	5.08±0.64^{ab}	4.10±0.47^{a}	5.02±0.48^{ab}	4.95±0.37^{ab}

[&]The data is the mean, and standard deviation, of three independent experimentations.

*Means on the same line with a letter (as superscript) in common are not significantly different. When no letter is indicated in a line of the table, it means that all values are statistically equivalent.

[#]n.d.: non detected.

Abbreviations used in order of appearance: Qui: quinic acid; OxHTY: oxidized hydroxytyrosol; HTY: hydroxytyrosol; TY: tyrosol; Van: vanillic acid; DEA: decarboxymethyl elenolic acid; *p*-Cou: *p*-coumaric acid; Fer: Ferulic acid; DesoxyEA: desoxyelenolic acid; HEA: hydroxyelenolic acid; EA: elenolic acid; HTY AcyE: Hydroxytyrosol acyclodihydroelenolate; HDOle Agly: hydroxy-decarboxy-methyloleuropein aglycone; Ole: oleuropein; Ole Agly: oleuropein aglycone; DOA: decarboxymethyl oleuropein aglycone (DOA isomer 2 has been named olecantahlic acid by other authors); Lut: luteolin; Pin: pinoresinol; 10-H Ole Agly: 10-hydroxy oleuropein aglycone; DLA: decarboxymethyl ligstroside aglycone (or oleocanthal); Api: apigenin; Dio: diosmetin; Lig Agly: ligstroside aglycone; MA: maslinic acid; BA: betulinic acid; OA: oleanolic acid.
